# A theory-driven user-centered design framework for wellness applications targeting young adults: The MiCARE methodology

**DOI:** 10.1177/20552076261436271

**Published:** 2026-03-31

**Authors:** Ayesha Thanthrige, Nilmini Wickramasinghe

**Affiliations:** 1 School of Computing, Engineering and Mathematical Sciences, 2080 La Trobe University, Melbourne, Australia

**Keywords:** Wellness engagement, digital health, user-centered design, theory-driven development, prevention

## Abstract

**Background:**

Lifestyle medicine is a rapidly growing field focused on preventing, managing, and, in some cases, reversing chronic diseases. Digital wellness applications offer opportunities to support and scale lifestyle medicine practices. However, most commercially available applications lack evidence-based design and theoretical grounding. Additionally, many compromise personalization and cultural relevance, which leads to poor sustained engagement, especially among young adults. Therefore, input from both end users and domain experts is vital to ensure responsible design and inform the design of user engagement features.

**Objective:**

This study outlines the systematic development of a design framework and feature blueprint for an AI-augmented wellness web app to support engagement and understanding among young adults. It also documents the process for digital health researchers and developers.

**Methods:**

This formative user experience design study used a three-phase user-centered design process. Phase 1 involved background analysis and conceptualization via systematic literature review, expert consultations, formative research with young adults, and rapid user interface/user experience design review. Phase 2 focused on wireframe development and evaluation through Figma prototypes with iterative expert feedback. Phase 3 covered prototype development. Conducted in Victoria, Australia, from April 2024 to October 2025, the process included an iterative feedback loop.

**Results:**

Phase 1 revealed barriers like high dropout rates, poor personalization, and cultural issues. Feedback from experts and users (60% female (mean age 27.3 years) and 40% male (mean age 28.4 years)) yielded five objectives: empathy-driven interaction, equity-focused accessibility, culturally responsive personalization, incremental goal setting, and intuitive onboarding. Preliminary user preferences for grid layouts (55%), large buttons (65%), and minimalist designs (50%) shaped features. A rationale table maps theory to features.

**Conclusion:**

This study presents a theory-driven, stakeholder-informed method for designing AI-augmented wellness app features guided by inclusive principles, while providing a replicable methodology for developing the design of engagement-oriented digital health solutions for young adults. The functional prototype requires rigorous usability testing to validate these preliminary design specifications.

## Introduction

### Background

Mobile health (mHealth) technologies—encompassing smartphones and IoT (internet of things) devices such as wearables and sensors—have become central to wellness promotion. With 7.3 billion smartphone subscriptions worldwide^1^ and 88% smartphone ownership in Australia,^2^ wellness applications represent a rapidly expanding digital health tool. The mHealth market is projected to grow from $87.6 billion in 2025 to $187.7 billion by 2033,^3^ with over 350,000 apps available globally addressing diet, physical activity, mental health, and chronic disease management.^4^

These technologies are particularly critical for young adults facing rising chronic disease risks, such as prediabetes, due to lifestyle factors.^5,6^ Mobile platforms enable self-monitoring and habit formation, with apps that track progress reinforcing sustained engagement through personalized experiences.^7^ Furthermore, mobile-responsive web applications offer cost-effective scalability, adaptive messaging, and real-time data collection.^8^

However, evidence-based design remains limited in commercial wellness applications.^9^ Studies show that many apps lack integration of behavioral theory,^10^ fail to incorporate accessibility standards,^11^ and demonstrate minimal stakeholder involvement in development.^12^ These gaps highlight the need for systematic, user-informed development processes.

The MiCARE application is a mobile-responsive progressive web application (PWA)—a web application that functions like a native mobile app, providing offline access and device integration—and leverages AI (e.g., chatbot with clinically verified responses, personalized recommendations and reminders) to address engagement and inclusivity gaps through personalization and accessibility.

### The potential for wellness apps in young adult health and wellness

Young adults (aged 18–34 years)^13^ navigate critical life transitions—higher education, employment, relationships, and identity formation^14^—that often coincide with increased vulnerability to chronic conditions. Prediabetes affects 374 million adults globally,^15^ characterized by elevated blood glucose levels^16^ reversible through early intervention.^17^ Sedentary behavior (averaging 8.8 h daily)^18,19^ and poor dietary habits^20^ compound these risks, yet young adults demonstrate low digital health engagement.^21,22^ Unlike acute care applications, chronic conditions require sustained self-management.^23^

Despite these risks, most wellness applications fail to address young adults’ unique motivational and contextual needs.^24^ Generic content and idealized imagery alienate diverse users, lacking the personalization and inclusivity critical for sustained engagement.^25^ Digital wellness solutions offer distinct advantages: flexible access to tailored tools and seamless integration into daily routines.^26^ These capabilities are particularly significant in preventive health contexts, where sustained self-management is essential for addressing chronic risks like prediabetes.

### Approaches to develop wellness apps

In the context of young adult wellness, few apps have been designed with input from young adults or experts in inclusive design. Consequently, there is a growing trend toward adopting user-centered design (UCD) approaches, particularly in digital health. UCD emphasizes collaborative design with users as active participants, ensuring that their needs, preferences, and lived experiences directly shape the final product.^27^ Despite this, reviews of commercial wellness applications reveal limited application of behavioral theory and inclusive design principles, which are associated with^10,28^ high attrition rates. A meta-analysis reported a 43% pooled dropout rate across mHealth interventions for chronic disease, with observational studies showing a 49% rate.^29^ For young adults, few apps incorporate their input or expert guidance on inclusivity, limiting relevance. However, apps with evidence-based features, such as personalized feedback, often show lower consumer popularity, suggesting collaboration between behavioral experts and commercial developers could enhance engagement.^30^ Wellness applications require social validity—the extent to which interventions are acceptable, appropriate, and meaningful to stakeholders—achievable through UCD approaches that co-design with users.^30^

### Theoretical foundation

This manuscript describes the formative user experience (UX) design phase of the MiCARE project, which involved developing the theoretical framework, conducting preliminary user research, creating wireframes, and building a functional prototype. Future phases will include systematic usability testing with diverse participants (planned Q2 2026) and iterative refinement based on empirical evaluation.

The MiCARE framework serves as the designed artifact—the tangible output of design science research methodology (DSRM)—synthesizing multi-theoretical models: Self-Determination Theory (SDT),^31^ the CARE framework (Compassion, Assistance, Respect, Empathy),^32,33^ UCD,^34,35^ and Inclusive Design.^36^ These theories were selected for their proven efficacy in digital health engagement and inclusivity, validated through stakeholder feedback. The Methods section details how these theories were operationalized through systematic stakeholder consultation and criterion-based feature selection.

The framework translates theoretical constructs into five design objectives (detailed in Methods): empathy-driven interactions, equity-focused accessibility, culturally responsive personalization, incremental goal setting, and intuitive onboarding. Post-deployment evaluation will employ Task-Technology Fit (TTF)^37^ and Unified Theory of Acceptance and Use of Technology (UTAUT)^38^ frameworks to assess usability and user acceptance.

Despite growing interest in wellness tools, the process of developing theory-informed, inclusive digital health solutions remains under-documented. This study addresses this gap by providing a systematic, replicable methodology for translating theoretical constructs into preliminary design specifications—a critical middle layer often absent in digital health literature.

## Methods

### Study design and context

This was a formative UX design study conducted in Victoria, Australia, between April 2024 and October 2025 (approximately 18 months). The study employed DSRM, focusing on developing and documenting a theory-driven design framework and functional wellness prototype. Health outcomes were explicitly excluded from this research scope as specified in the ethics protocol. Guiding principles for the MiCARE intervention were formulated at an early stage to provide a framework for making decisions during development (Textbox 1). These were based on evidence from systematic reviews of digital health engagement^10,39^ and UCD principles^9,12^ and were refined progressively as the intervention development proceeded based on development outcomes.

Textbox 1. Guiding principles for the MiCARE intervention

- Support sustained engagement across wellness domains with personalization and user autonomy.
- Promote accessibility, cultural relevance, and empathy through design.
- Reinforce motivation via feedback and gamification.
- Meet ethical requirements (UX-focused scope)

## Overview

**Figure 1.**
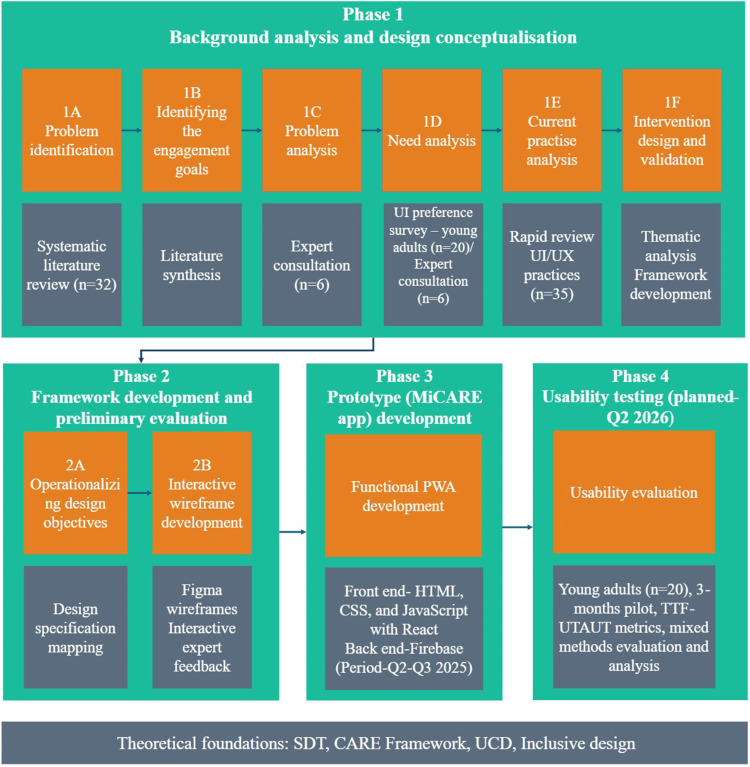
Overview of the multi-phase user-centered and design science process used to develop the MiCARE wellness application.

The MiCARE UX design development process followed three phases (Figure 1) consisting of (Phase 1) background analysis and design conceptualization, (Phase 2) wireframe development and preliminary evaluation, and (Phase 3) functional prototype development. An iterative feedback loop was applied throughout the design process to inform and refine the framework. This UCD approach aligns with established methodologies while extending Phase 1 with theoretically grounded intervention design.

### Phase 1: Background analysis and design conceptualization

#### Step 1A: Problem identification

A systematic literature review (SLR) following PRISMA guidelines searched four databases (PubMed, Scopus, CINAHL, and additional sources) for digital health interventions for T2DM and prediabetes (January 2016–October 2025). Thematic analysis of 37 studies identified key barriers and facilitators.^40^

#### Step 1B: Identifying the engagement goals based on literature

Rather than targeting a single health behavior, MiCARE was designed to support sustained wellness engagement across two main domains (i.e., diet and physical activity) which are two main pillars in lifestyle medicine. This broader engagement goal was validated through expert consultations and formative research. The “less is more” principle was applied to focus on foundational design elements that frame long-term use and build incrementally.^41^

#### Step 1C: Formative research with experts and users — problem analysis

A problem analysis was conducted to provide insight into problems experienced by users and identified by experts to determine intervention targets and set boundaries. Six experts (two female and four male) with over 10 years of experience in digital health, technology development (including UI/UX), and clinical practice were consulted to review preliminary design objectives and provide feedback on feasibility and cultural appropriateness. Expert feedback was collected via email using open-ended questions and structured feedback surveys aligned with the topics listed in Table 1.

**Table 1.** Topics explored with experts and young adult users.

Participant group	Topics explored
Experts (*n* = 6)	Usability and accessibility considerations Visual design preferences (colour schemes, contrast, fonts) UI recommendations aligned with Web Content Accessibility Guidelines (WCAG 2.1 AA) Gamification elements and motivation strategies Representation, Cultural nuances and inclusive imagery Feasibility of development and deployment Relevance to self-management and health education Evaluation and validation methods
Young adults (*n* = 20)	Dashboard layout intuitiveness Screen interaction ease Screen clutter perception (cognitive/information load) Personalization freedom in UI design Preferred interaction style for wellness applications

Experts were purposively selected based on their availability to provide structured feedback within the study timeline. The expert panel (*n* = 6) comprised a Senior Clinician, a Professor in Digital Health, an Associate Professor in Computer Science, and three Research Fellows in Computer Science. Experts were recruited through the research team's professional networks within La Trobe University.

#### Step 1D: Needs analysis

An additional needs analysis was performed to further investigate specific preferences. Preliminary co-design was conducted with 20 young adults (60% female [mean age 27.3], 40% male [mean age 28.4]). Participants were recruited via advertisements at La Trobe University and email invitations to students. Recruitment followed a self-selection process. A purposive sampling approach was then applied at the eligibility stage, with participants included based on predefined inclusion and exclusion criteria aligned with the study objectives and ethics approval.

Inclusion criteria required participants to be young adults aged 18–34 years, able to read and understand English, with no compulsive exercise behaviours or history of disordered eating, and willing to provide informed consent. Individuals who did not meet these criteria were excluded during screening. At this stage, participants were not provided with the app; instead, they completed a structured survey using visual wireframes that presented different layout options and interaction styles. A structured user interface (UI) feedback survey was distributed to gather initial insights on UI preferences. The survey included questions with visual design options and open-ended items exploring topics listed in Table 1. Sample sizes were informed by established usability testing guidelines,^42,43^ and qualitative saturation guidelines for exploratory studies^44^ which suggest that 15–20 participants can identify ∼90% of usability issues. Hence, we recruited *n* = 20 to ensure adequate representation across the five design preference categories explored. This exploratory sample is suitable for identifying directional preferences and generating hypotheses for wireframe development.

#### Step 1E: Current practice analysis

A rapid review following PRISMA principles searched six databases (IEEE Xplore, PubMed, Web of Science, ACM Digital Library, CINAHL, Scopus) for empirical studies (2020–2025) reporting UI features, usability outcomes, or design preferences in digital health applications. Grey literature and non-English studies were excluded.

Two reviewers independently screened studies using Covidence. Data from 35 included studies were synthesized thematically using NVivo.

#### Step 1F: Intervention design

Qualitative data from expert and user feedback were analyzed using theory-guided thematic synthesis. Initial codes (e.g., autonomy, empathy) were deductively derived from SDT, CARE, UCD, and Inclusive Design, with inductive codes added for emergent themes (e.g., cultural relevance) using NVivo software. Descriptive statistics were calculated in SPSS to summarize participant demographics and UI preferences (e.g., means, frequencies, and percentages).

A criterion-based selection process was used to determine which preliminary user preferences would be integrated into the framework. Preferences were included if they met three conditions: (1) aligned with at least one theoretical construct, (2) supported by expert consensus, and (3) feasible within the scope of MiCARE's development.

Each objective was mapped to its app features in the next phase.

### Phase 2: Wireframe development and preliminary evaluation

#### Step 2A: Operationalizing design objectives

The analytical process translated refined engagement goals into corresponding design objectives. Each design objective was mapped to its theoretical foundation, stakeholder input, and operational features in a design rationale table to document the complete translation process from theory to user-informed design specifications.

#### Step 2B: Interactive wireframe development

Interactive wireframes were developed using the Figma tool to visualize key features informed by the design rationale table. The wireframes served as a critical step in translating theory into practice, enabling iterative refinement based on expert feedback. The wireframes focused on operationalizing the five design objectives: empathy-driven interaction, equity-focused accessibility, culturally responsive personalization, incremental goal setting, and intuitive onboarding.

Wireframes were iteratively refined based on expert review, with design changes logged with rationale. Expert feedback informed refinements to enhance usability, emotional resonance, and cultural inclusivity, including visual design updates, accessibility considerations, and navigation streamlining.

### Phase 3: Prototype development

Following the completion of Phase 2, a functional PWA prototype was developed by the lead researcher based on the design rationale specifications and expert-validated wireframes. The prototype was built using a modern web technology stack: HTML, CSS, and JavaScript with React for the frontend, and Firebase for the backend. This architecture is suitable for both iOS and Android platforms, enabling future scalability and cost-effective maintenance. Development was conducted over a six-month period (Q2-Q3 2025) with self-managed biweekly sprint cycles.

The development process involved translating the design rationale table (Table 2) into functional components. The empathetic chatbot was developed using Google Dialogflow, with response templates that were validated by the senior clinician from the expert panel to ensure clinical accuracy and appropriate empathetic tone aligned with the CARE framework principles. Key technical implementation decisions included using responsive design frameworks to ensure optimal performance across devices, implementing accessibility features using built-in accessibility APIs and WCAG specifying contrast ratios, font sizes, and interactive element requirements. —Compliance standards, developing a secure authentication system with privacy-by-design principles following approved university ethics protocols, and creating a cloud-based backend infrastructure for data management using Firebase Firestore. All technical architecture decisions were discussed and finalized with the research supervisors to ensure alignment with ethical requirements and scalability considerations.

**Table 2.** Mapping of design objectives to theoretical models and feature logic.

Design objective	Supporting theory	User preference/expert feedback	Proposed features
Empathy-driven interactions	SDT, CARE	“I want the app to feel like a friend, not a coach.”	Empathetic chatbot with clinically verified responses and affirming tone; personalized reminders with supportive language; empathetic feedback
Equity-focused accessibility	Inclusive Design, CARE	“Colour blindness affects 8% of males—design must be WCAG compliant.”	Two-tone colour scheme (dark blue/white), dyslexia-friendly fonts, WCAG-compliant contrast and typography, and multilingual interface support.
Culturally responsive personalization	SDT, Inclusive Design	“Avoid idealized body imagery… Use symbolic visuals.”	Symbolic visuals (e.g., shoes for walking, vegetables for nutrition); diverse avatar options representing various body types; partial-body imagery (hands, legs) instead of full-body idealized images; customizable visual preferences
Incremental goal setting	SDT	“Start with simple wins… build confidence.”	Dynamic goal setting module with three difficulty levels (easy, medium, hard) for diet and physical activity; gamification elements (points, badges, rewards); visual progress tracking with celebratory feedback
Intuitive onboarding	UCD, UTAUT	“Implement a guided tour… Keep layout simple and clear.”	Guided video tour introducing key features; learning hub with educational content; minimalistic dashboard with grid-based tiles; customizable modules for personalized at-a-glance view; reduced click paths

The hands-on development approach by the lead researcher enabled translation of theoretical constructs into functional features while maintaining close alignment with the MiCARE framework throughout implementation. Regular consultation with the research supervisor and periodic technical reviews with members of the expert panel ensured quality assurance and theoretical fidelity throughout the development process.

### Phase 4: Usability testing

Systematic usability testing (Planned for Q2 2026) of the completed functional prototype will be conducted with young adults (*n* = 20) in Victoria, Australia, including participants from diverse cultural backgrounds in both metropolitan and regional areas. A three-month pilot evaluation will employ an integrated TTF ^37^ and UTAUT ^38^ framework through ethics-approved surveys administered at three timepoints (initial, mid-point, final) to assess usability, perceived usefulness, and satisfaction. Self-reported metrics will be triangulated with real-time usage analytics extracted from Firebase, including usage patterns, feature interactions, and navigation patterns. Qualitative feedback may be analyzed using machine learning-assisted methods, while quantitative data will undergo descriptive and inferential statistical analysis.

## Ethical considerations

Ethical approval was obtained from La Trobe University's Human Research Ethics Committee (HEC24507). Written informed consent was obtained from all participants before study participation. Participant data were coded and stored securely in accordance with university ethics and data management requirements. The study focused exclusively on UX outcomes—usability, usefulness, and satisfaction—and did not assess clinical health outcomes.

## Results

### Phase 1: Background analysis and design conceptualization

#### Step 1A: Problem identification

Thematic synthesis identified key barriers: high dropout rates^40^ and low risk perception undermining autonomy,^45–48^ inadequate personalization leading to declining usage,^24,49,50^ environmental constraints (socioeconomic barriers, complex interfaces),^16,51,52^ cultural and language barriers.^53,54^ and low digital literacy.^55,56^ Facilitators included cultural tailoring enhancing relevance,^55^ personalized feedback supporting motivation,^53,57,58^ user-friendly design improving accessibility, and peer support fostering community engagement.^53,59,60^

#### Step 1B: Identifying the engagement goals based on literature

Solutions enabling user-defined goals and personalized feedback showed higher retention,^54^ while rigid, behavior-specific solutions (e.g., calorie tracking) were linked to higher dropout.^48^ Solutions framing wellness as holistic and self-directed sustained greater interest, informing survey design (Table 1).

Based on the findings, MiCARE's initial engagement goals were defined as follows:

- Support sustained engagement across wellness domains (e.g., diet and physical activity)
- Enable user-defined goal setting and dynamic difficulty adjustment
- Reinforce motivation through personalized feedback and gamification
- Promote emotional safety and cultural relevance in all interactions

These goals were further validated through expert consultations and formative user research, ensuring alignment with both theoretical models and lived experience.

#### Step 1C: Formative research with experts and users — problem analysis

Formative research was conducted to refine MiCARE's engagement goals and validate design priorities through input from both experts and young adult users. This process involved structured feedback from six experts in digital health, alongside a UI preference survey completed by 20 young adults.

### Expert feedback

Expert feedback identified six priority themes (Table 3).

**Table 3.** Expert feedback on engagement goals and design priorities.

Theme	Sub theme	Example quotes
Autonomy & personalization	User-defined goals, flexible tracking	“Maybe you can add a goal of helping users break bad habits and promote good habits. … Let users choose what matters to them”
Incremental platform	Small goals that grow over time	“Incorporate small, achievable goals that grow incrementally. e.g., week 1→ 5-min walks or a single glass of water in the morning. Week 2→ Increase duration or frequency”
Ease of use & onboarding	Guided tour, minimalistic layout	“Minimalistic design ensuring clear navigation. … The solution is generally easier to use and intuitive. It minimizes number of clicks so is also efficient and user friendly”
Gamification & motivation	Points, badges, progress bars	“Use gamification elements to celebrate achievements (use rewards, badges)”
Broader wellness dimensions	Diet, physical activity	“Young adults want holistic wellness rather only single aspect e.g., weight management”
Accessibility & inclusion	WCAG compliance, diverse visuals	“Use a two-contrasting-colour scheme (e.g., black and white, dark blue and white, or dark green and white) for better design consistency. …” “Consider accessibility colour blindness affects about 8% of males and approaching 1% of females. Refer to Web Content Accessibility Guidelines (WCAG)”

### Preliminary user feedback

The structured UI Feedback Survey presented participants with visual wireframes illustrating five key design decisions. Each item displayed three systematically varied options (A, B, C) differing in layout complexity, information density, and interaction style. For example, the dashboard layout question offered: (A) a grid-based tile system with 6–8 large tiles, (B) horizontal tabs with left-to-right content organization, and (C) a vertical scrolling feed. Participants selected their preferred option and provided open-ended rationale. This approach enabled exploration of preferences at the formative stage without requiring interaction with a functional prototype.

The UI Feedback Survey revealed four overarching preference themes among young adult participants:

- Intuitive and clear navigation (participants favoured streamlined layouts)
- Ease of interaction (straightforward, low-effort screen designs)
- Minimalist and uncluttered aesthetics (reduced cognitive load)
- Personalization and user control (flexibility in customizing the experience)

Quantitative preferences across the five design elements are summarized in Table 4. Overall, participants showed strong preferences for simpler, less cluttered interfaces with large-button interactions. These preliminary findings informed early wireframe development and will be validated through objective usability testing in Phase 4.

**Table 4.** Preliminary UI preferences from young adult participants.

Design element	Option A	Option B	Option C
Dashboard Layout	55% (*n* = 11) Grid based tiles	25% (*n* = 5) Horizontal tabs	20% (*n* = 4) Vertical feed
Interaction Style	55% (*n* = 11) Interactive slider with emoji	30% (*n* = 6) Dropdown menus	15% (*n* = 3) Checkboxes
Visual Density	50% (*n* = 10) Swipe carousel	35% (*n* = 7) Stacked vertical list	15% (*n* = 3) Dropdown menus
Personalization	55% (*n* = 11) Visual customization (choose avatar, dark mode -enable/disable)	25% (*n* = 5) Detailed settings dropdown	20% (*n* = 4) Simplified toggles (on/off)
Action Patterns	65% (*n* = 13) Large action buttons	25% (*n* = 5) Scrollable carousels	10% (*n* = 2) Collapsible dropdowns

Initial quantitative preferences from 20 young adult participants revealed:

Overall, initial user feedback showed clear preferences for simpler, less cluttered designs with large button interaction styles. These initial preferences informed early wireframe design and will be validated through systematic usability testing in subsequent phases.

#### Step 1E: Current practice analysis

The rapid review of 35 studies identified evidence-based design elements to enhance accessibility, engagement, and inclusivity: larger touch targets (≥20 mm) improving accuracy,^61^ high-contrast colour schemes enhancing readability with preferences for skeuomorphic designs— A design style in UI that imitates the appearance, textures, shadows, and visuals of real-world physical objects (e.g., buttons that look like actual 3D buttons, gradients, and realistic shadows) in ePHR (electronic personal health record) systems,^62^ grid-based layouts reducing task completion time,^63,64^ simplified interactions minimizing cognitive load,^62,65^ customizable fonts boosting satisfaction,^66,67^ and minimalist designs improving engagement.^68,69^

These evidence-based insights align with MiCARE's design decisions to prioritize accessibility and usability.

We implemented primary action targets of ≥48 × 48 pixels to reduce touch errors,^61,65^ adopted grid-based tiles with clear visual hierarchies for faster recognition,^70,71^ utilized high-contrast two-tone palettes compliant with WCAG standards,^62,72^ and incorporated swipe carousels to minimize cognitive load.^67,69^ This current practice analysis served as moderating factors in the design rationale table, ensuring MiCARE's features were grounded in contemporary UI/UX research while addressing identified gaps.

#### Step 1F: Intervention design

Based on the synthesis of expert and user feedback, MiCARE's engagement goals were refined from the initial literature-based goals (Step 1B) to include:

- Support sustained engagement across multiple wellness domains considered (physical activity, diet)
- Enable user-defined goal setting with dynamic difficulty adjustment
- Reinforce motivation through personalized feedback, gamification, and visual progress tracking
- Promote emotional safety, cultural relevance, and inclusive design
- Ensure accessibility through WCAG compliant design

These refined goals served as the foundation for developing the intervention design.

### Phase 2: Wireframe development and preliminary evaluation

#### Step 2A: Operationalizing design objectives

The analytical synthesis translated the five refined engagement goals into five corresponding design objectives:

- Empathy-driven interactions (derived from “Promote emotional safety…”)
- Equity-focused accessibility (derived from “Ensure accessibility through WCAG…”)
- Culturally responsive personalization (derived from “Promote…cultural relevance and inclusive design”)
- Incremental goal setting (derived from “Enable user-defined goal setting with dynamic difficulty adjustment”)
- Intuitive onboarding (derived from “Support sustained engagement…” combined with minimizing cognitive load)

Each design objective was then mapped to its theoretical foundation, stakeholder input, and operational features in a design rationale table (Table 2). This table documents the complete translation process from theory to user-informed design specifications, forming the MiCARE framework.

**Figure 2.**
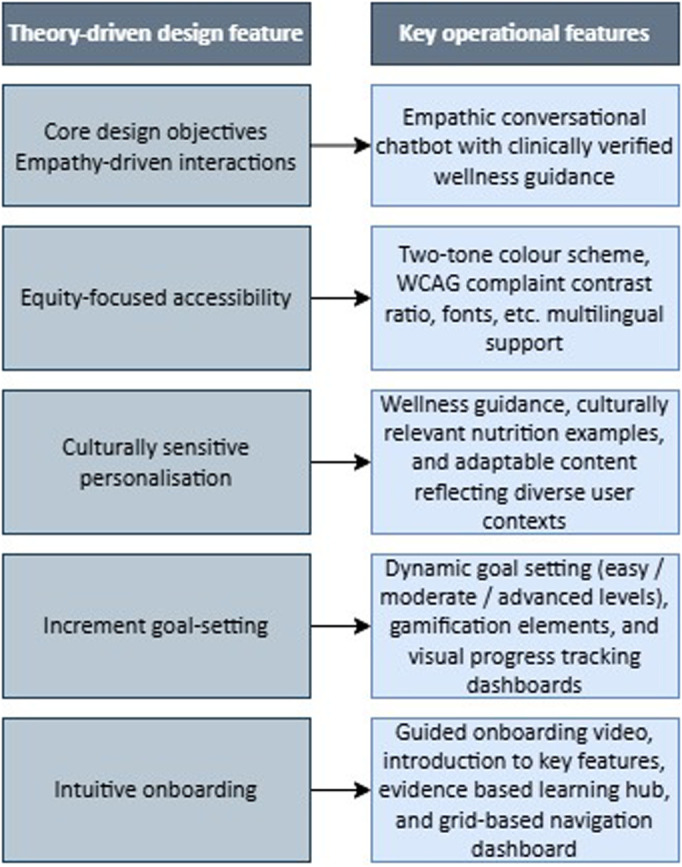
Mapping of core design objectives derived from theoretical models to operational application features.

Figure 2 presents a visual overview of MiCARE's theory-driven design features.

These design objectives form the foundation of the MiCARE framework and inform the translation of design objectives into app features (Step 2B).

#### Step 2B: Interactive wireframe development

The design rationale table (Table 2) guided translation of design objectives into features operationalizing SDT constructs (autonomy, competence, relatedness) and CARE principles (compassion, empathy). Expert recommendations informed accessibility decisions aligned with WCAG standards. “Use two contrasting colours like dark blue and white. It's easier to read and more inclusive.”

“Make sure the app works for colour blind users. That's 8% of males, don’t leave them out.”

The onboarding experience was designed to be intuitive and emotionally supportive. A guided video tour introduces key features, while the dashboard layout minimizes cognitive load and maximizes glanceability. These decisions were reinforced by user feedback emphasizing clarity, simplicity, and autonomy.

“If it looks messy, I won’t use it. I need clarity.”

“Let me choose what matters—don’t assume I want to count steps.”

Together, these features operationalize the MiCARE framework in a way that is theoretically coherent, and user informed. They provided a robust foundation for wireframe development and future usability testing.

### Wireframe refinement

**Figure 3.**
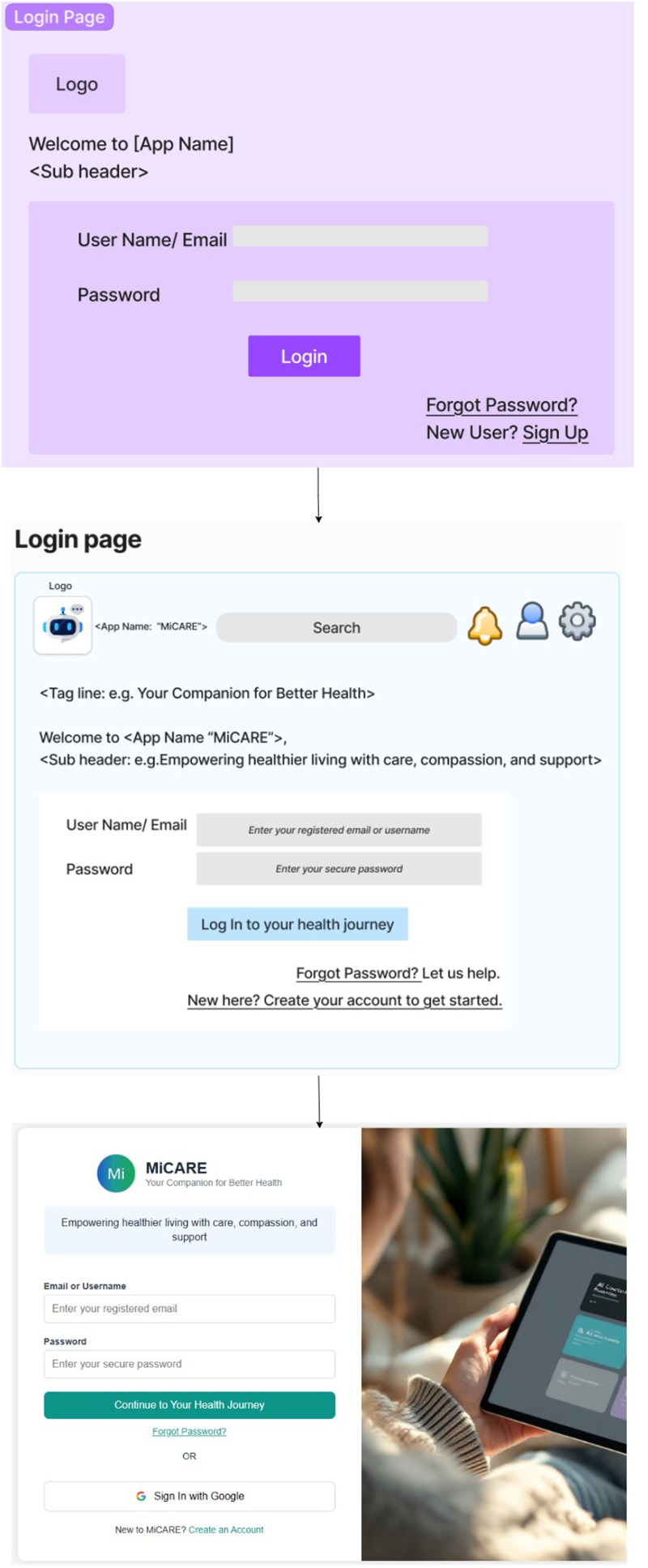
Evolution of the login and onboarding interface from initial wireframe to the refined prototype design.

Interactive wireframes were developed using the Figma platform to visualize key features. Figure 3 illustrates iterative refinement of the MiCARE login interface.

Initial designs featured a monochromatic purple palette and standard dashboard layout. However, iterative expert feedback prompted several refinements to enhance usability, emotional resonance, and cultural inclusivity.

“The shades of light purple look dull. Consider using colours the human eye is naturally used to—blue, white, green, black.”

“Use two contrasting colours like dark blue and white. It's easier to read and more inclusive.”

In response, the visual design was updated to a two-tone scheme (dark blue and white), improving contrast and readability. Accessibility considerations were integrated using WCAG compliant colour palettes and dyslexia-friendly fonts. Symbolic and partial-body imagery replaced idealized visuals to promote psychological safety and representation.

“Avoid idealized or overly fit body images… Use symbolic objects or partial body visuals like hands and legs.”

The onboarding experience was redesigned to include a guided video tour and glanceable dashboard, minimizing cognitive load and supporting early engagement. Navigation was streamlined to reduce click paths and improve flow.

“The wireframe design is generally easier to use and intuitive. It minimizes clicks and feels emotionally safe.”

Key features in the wireframes included:

- Learning hub providing educational content on diet and physical activity
- Empathetic chatbot with clinically verified responses
- Customizable goal setting module with dynamic difficulty levels (easy, medium, hard) for diet and physical activity
- Gamification elements including rewards, badges, and progress tracking
- Personalized reminders and recommendations for diet and physical activity
- Progress dashboard showing achievements
- Symbolic visuals and avatars representing diverse body types
- Minimalistic layout with clear navigation

Wireframes were iteratively refined based on expert review. Suggestions for further refinement included:

“Instead of ‘New here? Create your account to get started,’ just say ‘Create New Account.’ It's cleaner.”

“Use sentence case—some people struggle with reading all capitals.”

The expert-validated wireframes reflect a synthesis of theoretical rigor, expert insight, and preliminary user-centered input. They provided a strong foundation for prototype development (Phase 3) and future usability testing with end users (Phase 4).

#### Phase 3: Prototype development

**Figure 4.**
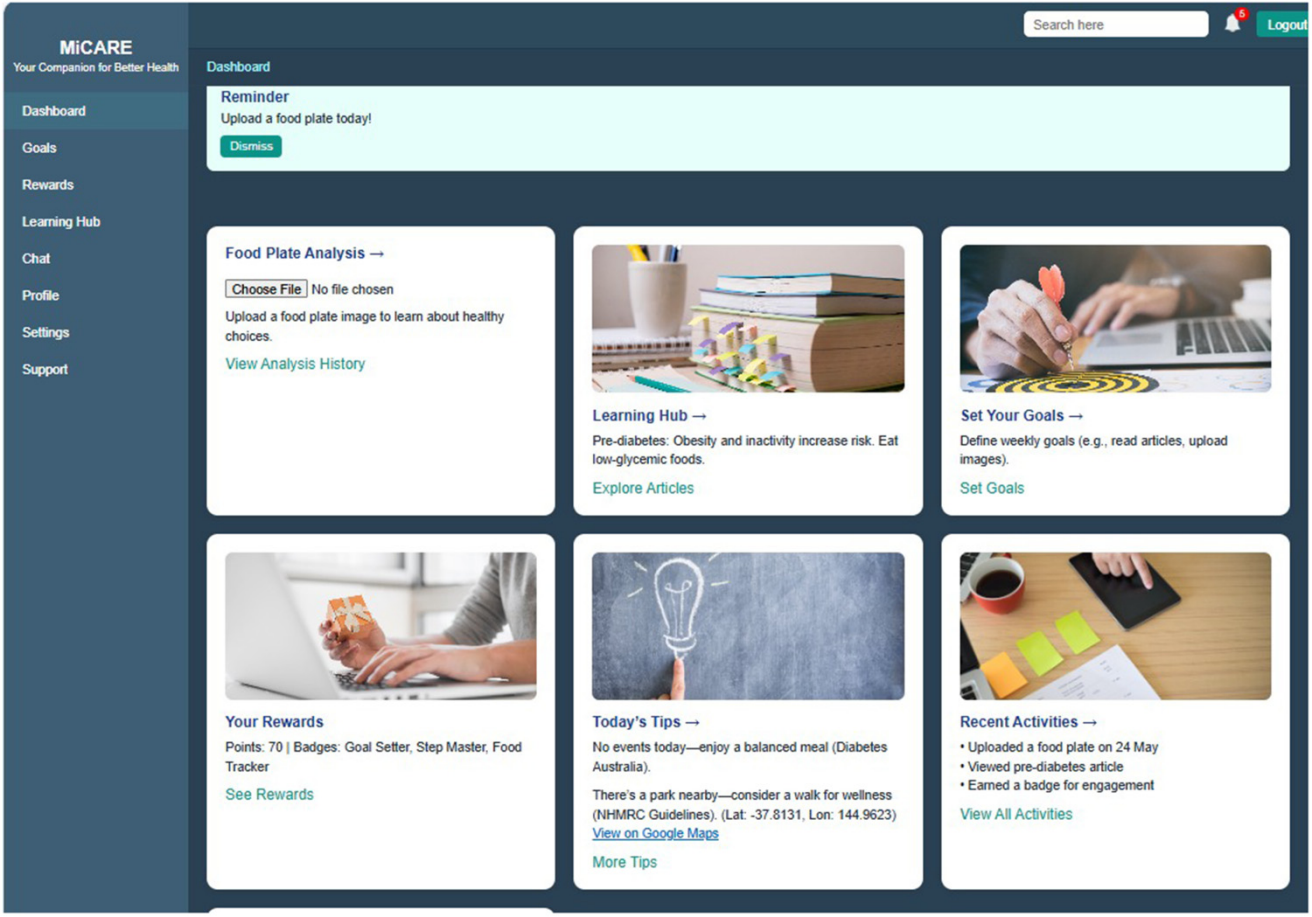
Screenshot of the functional PWA showing the main dashboard and feature modules.

The development process resulted in a functional PWA implementing six key features from Table 2: empathetic chatbot (Google Dialogflow with clinically verified responses), learning hub, customizable goal-setting module, gamification elements (points, badges, progress tracking), personalized reminders, personalized recommendations, and progress dashboard (see Figure 4). Technical implementation prioritized WCAG accessibility compliance and secure authentication. The chatbot's conversational flow was iteratively refined based on senior clinician feedback to balance empathy with clinical accuracy. The completed prototype is ready for deployment on a secure testing environment, accessible via web browsers across desktop, tablet, and mobile devices, in preparation for Phase 4 usability evaluation.

## Discussion

### Principal findings

Despite the rapid proliferation of wellness applications, few have been developed through systematic, theory-driven, and UCD processes. Most commercial solutions continue to emerge in isolation from evidence-based frameworks, limiting their capacity to promote sustained engagement, inclusivity, and cultural relevance among diverse young adult populations. This study developed a design framework for an inclusive, AI-augmented wellness application targeting young adults. The present work represents a formative stage of UX design, establishing theoretical foundations and preliminary feature specifications culminating in a completed functional prototype.

Following a three-phase DSRM that focuses on creating and evaluating artifacts (frameworks, prototypes) to solve identified problems, incorporating a SLR, expert consultations, rapid review of current UI/UX practices, and formative research with young adults, we constructed a theoretically grounded framework integrating SDT, the CARE framework, UCD, and Inclusive Design principles.

The framework produced five core design objectives: empathy-driven interactions, equity-focused accessibility, culturally responsive personalization, incremental goal setting, and intuitive onboarding. These objectives were translated into prototype feature specifications, including an empathetic chatbot concept, WCAG-compliant UI principles, customizable goal modules for diet and physical activity, gamified rewards, and personalized reminders.

Preliminary user preferences (grid-based layouts 55%, large-button interactions 65%, minimalist designs 50%) informed feature specifications, though these require validation in Phase 4. The lead researcher's involvement throughout all phases ensured theoretical fidelity and eliminated translation gaps, while systematic stakeholder engagement (domain experts, clinicians, end users) provides a replicable methodology for UX-focused wellness tools.

However, it is essential to emphasize that this framework represents a conceptual foundation with a completed functional prototype that requires extensive validation through usability testing, and iterative refinement before any claims about user adoption or behavioral outcomes can be substantiated, as planned in subsequent research phases.

### Theoretical contributions and framework positioning

The MiCARE framework extends existing wellness design approaches by addressing gaps specific to digital UX design for young adults, contributing methodological rigor and increasing the likelihood of adoption.

Verstraeten et al.^73^ employed the Attitude-Social influence-self-Efficacy (ASE) model—a behavioral theory explaining how attitudes, social norms, and self-efficacy predict health behaviours model to identify eating behavior determinants (taste preferences, access barriers) in Ecuadorian adolescents. MiCARE operationalizes such determinants into interface features (e.g., customizable goal modules).

Joshanloo and Weijers^74^ used multidimensional scaling to map mental well-being dimensions across cultures (United States, Japan, Iran), identifying eudaimonic, hedonic, and existential-relatedness components. Their cross-cultural findings directly informed MiCARE's emphasis on culturally responsive personalization, ensuring that wellness features accommodate varying conceptualizations of health and well-being rather than imposing a single cultural framework. MiCARE bridges this by translating abstract well-being constructs into concrete UX elements (e.g., customizable goal modules and symbolic imagery).

Thumboo et al.^75^ identified 27 culturally relevant health domains through focus groups with Singaporean participants but did not propose technological implementation pathways. MiCARE addresses this translational gap by mapping comparable wellness domains (diet and physical activity) to specific digital features (e.g., learning hub content and customizable goal-setting modules), illustrating how culturally grounded conceptual frameworks can be operationalized in user-centered digital interventions.

Unlike the Behavioral Intervention Technology (BIT) model, which prioritizes behavior change mechanisms.^76,77^ MiCARE emphasizes UCD to address high app abandonment rates (>50% within 100 days) among young adults.^78^

### Practical contributions

This study offers actionable resources: a design rationale table (Table 2) enabling theory-to-implementation translation, a criterion-based selection process for feature prioritization, and a functional prototype demonstrating feasibility with a validated technology stack for wellness applications.

### Limitations

This study has limitations affecting its generalizability and scope. The sample size of 20 young adults was sufficient for this exploratory, formative UX design study aimed at identifying preliminary design preferences and generating hypotheses for wireframe development.^52–54^. However, this sample size and limited demographic diversity constrain claims of cultural inclusivity. Future research must recruit participants with greater demographic diversity to validate the framework's inclusivity empirically.

UI/UX preference data relied entirely on self-reported responses to hypothetical wireframes, without observed behaviour or objective usability metrics. Stated preferences may not reflect actual usage patterns or satisfaction with the functional prototype.

Finally, the framework assumes that selected features—such as symbolic imagery, dyslexia-friendly fonts, and WCAG compliant colour schemes—promote emotional safety and inclusivity. These choices are grounded in prior evidence and design principles,^61–72^ but their effectiveness in the MiCARE context remains untested empirically. The framework's applicability to other age groups or cultural settings is also unexamined. These limitations are characteristic of formative UX design studies and will be mitigated in Phase 4 through larger, more diverse recruitment and objective behavioral metrics.

### Future research directions

Future studies should prioritize systematic usability testing of the functional prototype with larger, more demographically diverse samples of young adults to empirically validate inclusivity claims and feature effectiveness. The next phase involves a three-month pilot evaluation (planned for Q2 2026) in Victoria, Australia, using TTF and UTAUT metrics to assess UX outcomes such as retention, navigation efficiency, and satisfaction. Data will be triangulated through surveys and real-time usage logs, providing objective behavioral insights to complement current self-reported preferences. Subsequent work should explore broader applicability across age groups and cultural contexts.

## Conclusions

The MiCARE framework integrates SDT, CARE, UCD, and Inclusive Design principles to guide development of wellness experiences for young adults prioritizing accessibility and cultural sensitivity. The completed prototype and replicable methodology serve as a practical template for evidence-based digital wellness interventions, requiring rigorous UX evaluation to substantiate real-world effectiveness.

## References

[1] Statista. Number of smartphone mobile network subscriptions worldwide from 2016 to 2025, with forecasts from 2025 to 2028 (in millions). 2025.

[2] Oviedo-Trespalacios O Nandavar S Newton JDA , et al. Problematic use of Mobile phones in Australia…is it getting worse? Front Psychiatry 2019; 10: 05. 10.3389/fpsyt.2019.00105 PMC6422909 30914975

[3] Mescher T Hacker RL Martinez LA , et al. Mobile health apps: guidance for evaluation and implementation by healthcare workers. Journal of Technology in Behavioral Science 2025; 10: 224–235.

[4] Koli PM Ketan . mHealth apps statistics and facts, 2025, https://market.biz/mhealth-apps-statistics-and-facts/ (accessed 5 October 2025).

[5] Wells C Spry C . An overview of smartphone apps. Can J Health Technol 2022; 2: 2. 37797157

[6] Leuzzi G Job M Scafoglieri A , et al. Smartphone apps and wearables for health parameters in young adulthood: cross-sectional study. JMIR Hum Factors 2025; 12: e64629. 10.2196/64629 PMC12407497 40902119

[7] Sousa Basto P Ferreira P . Mobile applications, physical activity, and health promotion. BMC Health Serv Res 2025; 25: 59. 40065339 10.1186/s12913-025-12489-z PMC11892298

[8] Przewięźlikowska A Ślusarczyk W Wójcik K , et al. Efficient and scalable architecture for location-based mobile applications using metrica. Autom Constr 2025; 172: 106056.

[9] Saparamadu A Fernando P Zeng P , et al. User-Centered design process of an mHealth app for health professionals: case study. JMIR Mhealth Uhealth 2021; 9: e18079. 10.2196/18079 PMC8088861 33769297

[10] Milne-Ives M Homer SR Andrade J , et al. Potential associations between behavior change techniques and engagement with mobile health apps: a systematic review. Front Psychol 2023; 14: 1227443. 37794916 10.3389/fpsyg.2023.1227443 PMC10545861

[11] Shin JH Shields R Lee J , et al. Quality and accessibility of home assessment mHealth apps for community living: systematic review. JMIR Mhealth Uhealth 2024; 12: e52996. 10.2196/52996 PMC10980499 38466987

[12] Koumpouros Y . User-centric design methodology for mHealth apps: the PainApp paradigm for chronic pain. Technologies 2022; 10: 25.

[13] (ACMA) ACaMA. The digital lives of younger and older Australians, 2022, https://www.acma.gov.au/publications/2022-01/report/digital-lives-younger-and-older-australians (accessed 5 October 2025).

[14] Fegert JM Gottschalk G Schneider R , et al. Navigating life transitions and mental wellbeing in the digital age: a call for stakeholders to embrace innovation and collaboration. Child Adolesc Psychiatry Ment Health 2025; 19: 67. 40517237 10.1186/s13034-025-00932-2 PMC12166554

[15] International Diabetes Federation. Diabetes facts and figures, 2021, https://www.idf.org (accessed 03/12/2024).

[16] Brzan PP Rotman E Pajnkihar M , et al. Mobile applications for control and self management of diabetes: a systematic review. J Med Syst 2016; 40: 210–210. 27520615 10.1007/s10916-016-0564-8

[17] An X Zhang Y Sun W , et al. Early effective intervention can significantly reduce all-cause mortality in prediabetic patients: a systematic review and meta-analysis based on high-quality clinical studies. Front Endocrinol 2024; 15: 1294819. 10.3389/fendo.2024.1294819 PMC10941028 38495794

[18] Loyen A Clarke-Cornwell AM Anderssen SA , et al. Sedentary time and physical activity surveillance through accelerometer pooling in four European countries. Sports Medicine (Auckland) 2017; 47: 1421–1435. 10.1007/s40279-016-0658-y PMC5488150 27943147

[19] Australia NHFo. Sit less, move more, https://www.heartfoundation.org.au/healthy-living/physical-activity/sit-less-move-more.

[20] Welfare AIoHa. Poor diet in adults, 2023, https://www.aihw.gov.au/reports-data/food-nutrition/poor-diet-in-adults (accessed October 5 2025).

[21] Andes LJ Cheng YJ Rolka DB , et al. Prevalence of prediabetes among adolescents and young adults in the United States, 2005-2016. JAMA Pediatr 2020; 174: e194498. 10.1001/jamapediatrics.2019.4498 PMC6902249 31790544

[22] Wu Y Zhang J Ge P , et al. Application of chatbots to help patients self-manage diabetes: systematic review and meta-analysis. J Med Internet Res 2024; 26: e60380. 10.2196/60380 PMC11653048 39626235

[23] Woodward A Walters K Davies N , et al. Barriers and facilitators of self-management of diabetes amongst people experiencing socioeconomic deprivation: a systematic review and qualitative synthesis. Health Expect 2024; 27: e14070. 10.1111/hex.14070 PMC11096776 38751247

[24] Lim SL Juan Tay MH Ong KW , et al. Association between Mobile health app engagement and weight loss and glycemic control in adults with type 2 diabetes and prediabetes (D’LITE study): prospective cohort study. JMIR Diabetes 2022; 7: e35039. 10.2196/35039 PMC9568822 36178718

[25] Nomura A Noguchi M Kometani M , et al. Artificial intelligence in current diabetes management and prediction. Curr Diab Rep 2021; 21: 61–61. 34902070 10.1007/s11892-021-01423-2 PMC8668843

[26] Aljuboori D Clary LK Alomairah SA , et al. Contextual adaptation of digital wellbeing interventions for young people: insights from a project in Saudi Arabia. Front Psychiatry 2024; 15: 1455962. 39957755 10.3389/fpsyt.2024.1455962 PMC11825761

[27] van Velsen L Ludden G Grünloh C . The limitations of user-and human-centered design in an eHealth context and how to move beyond them. J Med Internet Res 2022; 24: e37341. 10.2196/37341 PMC9582917 36197718

[28] Linardon J . Rates of attrition and engagement in randomized controlled trials of mindfulness apps: systematic review and meta-analysis. Behav Res Ther 2023; 170: 104421. 37862854 10.1016/j.brat.2023.104421

[29] Meyerowitz-Katz G Ravi S Arnolda L , et al. Rates of attrition and dropout in app-based interventions for chronic disease: systematic review and meta-analysis. J Med Internet Res 2020; 22: e20283. 10.2196/20283 PMC7556375 32990635

[30] Bear HA Ayala Nunes L Ramos G , et al. The acceptability, engagement, and feasibility of mental health apps for marginalized and underserved young people: systematic review and qualitative study. J Med Internet Res 2024; 26: e48964. 10.2196/48964 PMC11322694 39078699

[31] Ryan RM Deci EL . Self-Determination theory and the facilitation of intrinsic motivation, social development, and well-being. Am Psychol 2000; 55: 68–78. 11392867 10.1037//0003-066x.55.1.68

[32] Sinclair S McClement S Raffin-Bouchal S , et al. Compassion in health care: an empirical model. J Pain Symptom Manage 2016; 51: 193–203. 26514716 10.1016/j.jpainsymman.2015.10.009

[33] Sanjeewa R Iyer R Apputhurai P , et al. Empathic conversational agent platform designs and their evaluation in the context of mental health: systematic review. JMIR Ment Health 2024; 11: e58974. 10.2196/58974 PMC11420590 39250799

[34] Norman DA Draper SW . User centered system design : new perspectives on human-computer interaction. Hillsdale, NJ: Lawrence Erlbaum Associates, 1986.

[35] Jiang Q Deng L Zhang J , et al. User-Centered design strategies for age-friendly Mobile news apps. SAGE Open 2024; 14: 48.

[36] Clarkson PJ Coleman R Keates S , et al. Inclusive Design: Design for the Whole Population. 1st ed. 2003 ed. London: Springer London, 2003.

[37] Goodhue DL Thompson RL . Task-Technology fit and individual performance. MIS Q 1995; 19: 213–236.

[38] Venkatesh V Morris MG Davis GB , et al. User acceptance of information technology: toward a unified view. MIS Q 2003; 27: 425–478.

[39] Szinay D Jones A Chadborn T , et al. Influences on the uptake of and engagement with health and well-being smartphone apps. Systematic Review J Med Internet Res 2020; 22: e17572. 10.2196/17572 PMC7293059 32348255

[40] Thanthrige A Wickramasinghe N . Digital health solutions for type 2 diabetes and prediabetes: systematic review of engagement barriers, facilitators, and outcomes. JMIR Diabetes 2026; 11: e80582. 10.2196/80582 PMC12981377 41818488

[41] Korpershoek YJG Hermsen S Schoonhoven L , et al. User-Centered design of a Mobile health intervention to enhance exacerbation-related self-management in patients with chronic obstructive pulmonary disease (copilot): mixed methods study. J Med Internet Res 2020; 22: e15449. 10.2196/15449 PMC7324997 32538793

[42] Nielsen J Landauer TK . A mathematical model of the finding of usability problems (pp.206–213). New York, NY, USA: ACM, 1993.

[43] Turner BO Paul EJ Miller MB , et al. Small sample sizes reduce the replicability of task-based fMRI studies. Commun Biol 2018; 1: 62. 30271944 10.1038/s42003-018-0073-z PMC6123695

[44] Guest G Bunce A Johnson L . How many interviews are enough?: an experiment with data saturation and variability. Field Methods 2006; 18: 59–82.

[45] Bailey-Davis L Wood GC Cook A , et al. Communicating personalized risk of diabetes and offering weight reduction program choice: recruitment, participation, and outcomes. Patient Educ Couns 2021; 104: 1193–1199. 33097360 10.1016/j.pec.2020.10.017

[46] Zhang J Oh YJ Lange P , et al. Artificial intelligence chatbot behavior change model for designing artificial intelligence chatbots to promote physical activity and a healthy diet: viewpoint. J Med Internet Res 2020; 22: e22845–e22845. 10.2196/22845 PMC7557439 32996892

[47] Nanditha A Thomson H Susairaj P , et al. A pragmatic and scalable strategy using mobile technology to promote sustained lifestyle changes to prevent type 2 diabetes in India and the UK: a randomised controlled trial. Diabetologia 2020; 63: 486–496. 31919539 10.1007/s00125-019-05061-y PMC6997257

[48] Rohling M Kempf K Banzer W , et al. Prediabetes conversion to normoglycemia is superior adding a low-carbohydrate and energy deficit formula diet to lifestyle intervention-A 12-month subanalysis of the ACOORH trial. Nutrients 2020; 12: 2022. 32646010 10.3390/nu12072022 PMC7400892

[49] Lim SL Tay MHJ Ong KW , et al. Association between Mobile health app engagement and weight loss and glycemic control in adults with type 2 diabetes and prediabetes (D'LITE study): prospective cohort study. JMIR Diabetes 2022; 7: e35039. 10.2196/35039 PMC9568822 36178718

[50] Hussain W Grundy J. Advice for diabetes self-management by ChatGPT models: Challenges and recommendations. 2025. DOI:10.48550/arxiv.2501.07931.

[51] Wang SCY Nickel G Venkatesh KP , et al. AI-based diabetes care: risk prediction models and implementation concerns. NPJ digital Medicine 2024; 7: 36–36. 38361152 10.1038/s41746-024-01034-7 PMC10869708

[52] Green JB Crowley MJ Thirunavukkarasu S , et al. The final frontier in diabetes care: implementing research in real-world practice diabetes care. Diabetes Care 2024; 47: 1299–1310. 38907682 10.2337/dci24-0001

[53] LeSeure P Chin E Zhang S . A culturally sensitive Mobile app (DiaFriend) to improve self-care in patients with type 2 diabetes: development study. JMIR diabetes 2024; 9: e63393. 10.2196/63393 PMC11535794 39432893

[54] Ayers JW Poliak A Dredze M , et al. Comparing physician and artificial intelligence chatbot responses to patient questions posted to a public social Media forum. JAMA Intern Med 2023; 183: 589–596. 37115527 10.1001/jamainternmed.2023.1838 PMC10148230

[55] Mehraeen E Mehrtak M Janfaza N , et al. Design and development of a Mobile-based self-care application for patients with type 2 diabetes. J Diabetes Sci Technol 2022; 16: 1008–1015. 33840235 10.1177/19322968211007124 PMC9264443

[56] Yeh MC Lau W Keady CA , et al. Evaluation of feasibility and acceptability of a web-based diabetes prevention program (DPP) for diabetes risk reduction in Chinese Americans in New York city. Front Public Health 2023; 11: 1199746. 37333528 10.3389/fpubh.2023.1199746 PMC10272575

[57] Salari R Niakan Kalhori SR GhaziSaeedi M , et al. Mobile-Based and cloud-based system for self-management of people with type 2 diabetes: development and usability evaluation. J Med Internet Res 2021; 23: e18167–e18167. 10.2196/18167 PMC8209530 34076579

[58] Sun H Zhang K Lan W , et al. An AI dietitian for type 2 diabetes Mellitus management based on large language and image recognition models: preclinical concept validation study. J Med Internet Res 2023; 25: e51300–e51300. 10.2196/51300 PMC10667983 37943581

[59] Nayak A Vakili S Nayak K , et al. Use of voice-based conversational artificial intelligence for basal insulin prescription management among patients with type 2 diabetes: a randomized clinical trial. JAMA network Open 2023; 6: e2340232. 10.1001/jamanetworkopen.2023.40232 PMC10692866 38039007

[60] Frediani JK Bienvenida AF Li J , et al. Physical fitness and activity changes after a 24-week soccer-based adaptation of the U.S diabetes prevention program intervention in hispanic men. Prog Cardiovasc Dis 2020; 63: 775–785. 32603753 10.1016/j.pcad.2020.06.012 PMC8650220

[61] Yu N Ouyang Z Wang H , et al. The effects of smart home interface touch button design features on performance among young and senior users. Int J Environ Res Public Health 2022; 19: 2391. 35206579 10.3390/ijerph19042391 PMC8872557

[62] Zhang S Song J . An empirical investigation into the preferences of the elderly for user interface design in personal electronic health record systems. Front Digit Health 2024; 5: 1289904. 38348367 10.3389/fdgth.2023.1289904 PMC10859482

[63] Gomez-Hernandez M Ferre X Moral C , et al. Design guidelines of Mobile apps for older adults: systematic review and thematic analysis. JMIR Mhealth Uhealth 2023; 11: e43186. 10.2196/43186 PMC10557006 37733401

[64] Dekkers T Melles M Vehmeijer SBW , et al. Effects of information architecture on the effectiveness and user experience of web-based patient education in middle-aged and older adults: online randomized experiment. J Med Internet Res 2021; 23: e15846. 10.2196/15846 PMC7970227 33656446

[65] Gao Q Sun Q . Examining the usability of touch screen gestures for older and younger adults. Hum Factors 2015; 57: 835–863. 25957042 10.1177/0018720815581293

[66] Kramer JM Schwartz AE Davies DK , et al. Usability and reliability of an accessible patient-reported outcome measure (PROM) software: the pediatric evaluation of disability inventory–patient-reported outcome (PEDI–PRO). American Journal of Occupational Therapy 2021; 75: 1–10. 10.5014/ajot.2020.040733 PMC7784036 33399049

[67] Lu L Cochrane KA Kang J , et al. Why are there so many steps?”: improving access to blind and low vision music learning through personal adaptations and future design ideas. ACM Trans Access Comput 2023; 16: 22.

[68] Panigutti C Beretta A Fadda D , et al. Co-design of human-centered, explainable AI for clinical decision support. ACM Trans Interact Intell Syst 2023; 13: 21.

[69] Griffin AC Khairat S Bailey SC , et al. A chatbot for hypertension self-management support: user-centered design, development, and usability testing. JAMIA Open 2023; 6: ooad073. 10.1093/jamiaopen/ooad073 PMC10491950 37693367

[70] Srivastava A Kapania S Tuli A , et al. Actionable UI design guidelines for smartphone applications inclusive of low-literate users. Proceedings of the ACM on Human-Computer Interaction 2021; 5: 1–30.36644216

[71] Heijsters FACJ van Loon GAP Santema JMM , et al. A usability evaluation of the perceived user friendliness, accessibility, and inclusiveness of a personalized digital care pathway tool. Int J Med Inf 2023; 175: 105070. 10.1016/j.ijmedinf.2023.105070 37121138

[72] Barbosa NM Hayes J Kaushik S , et al. “Every website is a puzzle!”: facilitating access to common website features for people with visual impairments. ACM Trans Access Comput 2022; 15: 19.

[73] Verstraeten R Van Royen K Ochoa-Avilés A , et al. A conceptual framework for healthy eating behavior in Ecuadorian adolescents: a qualitative study. PLoS One 2014; 9: e87183. 10.1371/journal.pone.0087183 PMC3906122 24489865

[74] Joshanloo M Weijers D . A two-dimensional conceptual framework for understanding mental well-being. PLoS One 2019; 14: e0214045. 10.1371/journal.pone.0214045 PMC6436799 30917191

[75] Thumboo J Ow MYL Uy EJB , et al. Developing a comprehensive, culturally sensitive conceptual framework of health domains in Singapore. PLoS One 2018; 13: e0199881. 10.1371/journal.pone.0199881 PMC6023157 29953526

[76] Curtis KE Lahiri S Brown KE . Targeting parents for childhood weight management: development of a theory-driven and user-centered healthy eating app. JMIR Mhealth Uhealth 2015; 3: e69–e69. 10.2196/mhealth.3857 PMC4526951 26088692

[77] Mohr DC Schueller SM Montague E , et al. The behavioral intervention technology model: an integrated conceptual and technological framework for eHealth and mHealth interventions. J Med Internet Res 2014; 16: e146. 10.2196/jmir.3077 PMC4071229 24905070

[78] Kidman PG Curtis RG Watson A , et al. When and why adults abandon lifestyle behavior and mental health Mobile apps. Scoping Review J Med Internet Res 2024; 26: e56897. 10.2196/56897 PMC11694054 39693620

